# Aberrant Expression of SIRT6 and VNN1 in Peripheral Blood Monocytes of Children with Primary Nephrotic Syndrome and Its Diagnostic and Prognostic Values

**DOI:** 10.1155/2022/6880974

**Published:** 2022-09-15

**Authors:** Peitong Han, Xiaohong Xi, Xiaoying Yuan, Chunzhen Li, Ling Liu, Jieyuan Cui

**Affiliations:** Department of Nephrology and Immunology, Children's Hospital of Hebei Province, Shijiazhuang, Hebei Province 050031, China

## Abstract

**Objective:**

The objective is to explore the aberrant sirtuin-6 (SIRT6) and Vanin-1 (VNN1) protein expression in peripheral blood monocytes (PBM) of children with primary nephrotic syndrome (PNS) and its diagnostic and prognostic values.

**Methods:**

83 child patients with nephrotic syndrome (NS) and 65 healthy volunteers were enrolled in the study. The test of SIRT6 and VNN1 was performed by the Western blot. The receiver operator characteristic (ROC) curve was used to analyze the diagnostic and prognostic value of SIRT6 and VNN1 for child patients with NS. The logistic regression was used to analyze the association of SIRT6 and VNN1 with the prognosis of NS child patients.

**Results:**

SIRT6 in monocytes in the study group was inferior versus the control, while VNN1 outweighed it. The AUC of the combined detection of SIRT6 and VNN1 for the diagnosis of NS was 0.854, with a sensitivity of 80.0% and a specificity of 80.7%. The AUC of combined detection of SIRT6 and VNN1 for the prognosis of NS was 0.860, with a sensitivity of 84.6% and a specificity of 79.2%. The logistic regression analysis showed that less than 21.09 in SIRT6 was the number of risk factors for the prognosis of NS child patients (*P* < 0.05).

**Conclusion:**

SIRT6 and VNN1 are provided with diagnostic and prognostic values for NS.

## 1. Introduction

Primary nephrotic syndrome (PNS) in children is a group of clinical syndromes in which elevated glomerular basement membrane permeability and loss of large amounts of protein from urine are due to multiple factors [1, 2]. Relevant studies manifest that aberrant nicotinamide adenine dinucleotide-dependent protein deacetylase sirtuin-6 (SIRT6) is available to aggravate renal insufficiency, renal tubular injury, and renal fibrosis, which is closely associated with the occurrence of renal disease [3, 4]. The maintenance of normal renal function requires a great deal of energy, and most of that required for life activities is provided via synthesized adenosine triphosphate (ATP) through the mitochondria. Furthermore, mitochondrial dysfunction is available to affect the renal function of patients, leading to the occurrence of renal disease, while SIRT6 is available to prevent renal tubulointerstitial fibrosis via ameliorating mitochondrial dysfunction, implying that its aberrant expression is supposed to lead to mitochondrial dysfunction and aggravate renal function injury. Vanin-1 (VNN1) is a kind of glycosylated phospholipid adenoinositol-anchored hydrolase in epithelial cells, which is augmented in the liver and kidney [5]. It has been reported that VNN1 is available to repress the antioxidant capacity of platelets, aggravate the oxidative stress response of the body cells, or affect renal function [6, 7]. VNN1 is able to mediate oxidative stress on the body's cells and inflammation. However, its association with the occurrence of nephrotic syndrome (NS) and diagnostic value for this disease are not yet clearly reported. Therefore, this study was to explore SIRT6 and VNN1 in peripheral blood monocytes (PBM) of NS and their values in clinical diagnosis and prognosis, offering references for the clinical diagnosis and evaluation of this disease.

## 2. Materials and Methods

### 2.1. Clinical Data

From January 2019 to February 2021, 83 child patients with NS were selected for the study group. This study has been approved by the ethics committee of the Children's Hospital of Hebei Province, and informed consent was signed by the guardian of every subject. All of whom met the diagnostic criteria for NS in Evidence-based Guidelines on Diagnosis and Treatment of Primary Nephrotic Syndrome in Children (Trial) [8]. The course of disease of the child patients ranged from 4 months to 2 years, with an average of (0.93 ± 0.13) years. Pathological types: 49 cases of simple NS and 34 cases of nephritis. Clinical symptoms: 79 cases of proteinuria and 69 cases of edema, and the selection of 65 healthy volunteers served as the control. Inclusion criteria: ① under the age of 14; ② complete clinical data. Exclusion criteria: ① child patients with severe cardiovascular and cerebrovascular diseases; ② child patients with autoimmune diseases involving systemic lupus erythematosus and allergic purpura; ③ child patients with congenital NS; ④ child patients with other types of nephropathies; ⑤ child patients with combined tumor diseases. No distinct differences were found in gender and age between the two (*P* < 0.05), as proved in Table 1.

### 2.2. Methods

Protein determinations of SIRT6 and VNN1 in monocytes are as follows: the collection of 20 mL fasting venous blood was from child patients within 24 h after admission and children in the control, and the division of samples was into two parts. SIRT6 protein in the samples was determined, and dilution was with an equal volume of Hank solution, and the addition of 20 mL of Ficoll solution was with a density of 1.007 (all the Institute of Bioengineering, Chinese Academy of Medical Sciences). The adoption of a SORVALL cryogenic centrifuge (Kojun Instrument Co., Ltd., USA) was for centrifugation. The mononuclear cell was sucked from the milky layer and centrifuged again after washing with Hank's solution. After trypan blue staining, a cell culture was performed in Roswell Park Memorial Institute 1640 medium, and the addition of 80 *μ*L sodium dodecyl sulfate (SDS) and a phenylmethylsulfonyl fluoride mixture of (100 : 1) was made into each culture well. Placement of the culture plate was on the ice for lysis, and the shift of the cell lysis solution to an Eppendorf (EP) tube. After centrifugation, the shift of the protein solution was to a clean EP tube and the determination of the sample protein concentration was via the Bio-Rad QuantaPhase II radioassay enzyme-linked immunosorbent assay. The remaining proteins were added to a quarter volume of 5 × SDS loading buffer, boiled for 10 min, and stored in a refrigerator after cooling as a standby. The addition of 1 × SDS loading buffer was made into a 20 *μ*L protein sample, and centrifugation was carried out at the same rate. After collecting the supernatant, the equivalent protein was added to 12% sodium dodecyl sulfate polyacrylamide gel electrophoresis gel well, then electroblotted onto a Polyvinylidene fluoride membrane, and sealed with a phosphate buffer solution involving 5% skim milk powder. The addition of diluted rabbit anti-SIRT6 (Abcam, UK, 1 : 5000) after equilibrium was reached at temperature, and then the accretion of 5 mL of diluted goat anti-rabbit secondary antibody (1 : 5000) was for the reaction. Meanwhile, the adoption of the Leica Quantimet 550 DMRXA chemiluminescence and image analysis system was for analysis, and calculation was with the ratio of the target and the internal reference gray values. The determination of VNN1 was done via the same method as the control. Monoclonal rabbit antihuman VNN1 primary antibody and sheep anti-mouse VNN1 secondary antibody (all Abcam, Britain) were adopted.

### 2.3. Observation Indexes

(1) The test of SIRT6 and VNN1 in monocytes of patients in the study group and healthy subjects in the control was performed via Western blot, comparison, and then analysis their diagnostic value for NS. (2) The division of child patients was into the severe and the mild groups in line with the occurrence of complications (acute renal failure, thrombosis, electrolyte disturbance, infection, renal tubular dysfunction, etc.), analysis of the assessed values of SIRT6 and VNN1, and the severity of NS child patients. (3) The treatment of child patients was via referring to evidence-based Guidelines for Diagnosis and Treatment of Primary Nephrotic Syndrome in Children (Trial) [8], and the division into good prognosis (positive urinary protein <+++) and unpleasing prognosis (positive urinary protein ≥+++) was in line with the disease outcome after 4 weeks of treatment. An analysis of the predictive values of SIRT6 and VNN1 on the prognosis of child patients with NS and its association with an unpleasing prognosis was adopted.

### 2.4. Statistical Treatment

Application of SPSS24.0 software was to process the data, representation of enumeration data was as percent, and the comparison of the differences of groups was via *χ*^2^ test. Expression of the measurement data was as ($\bar{x}$ ± s) after the normal test. The comparison of the differences between groups was via the *t*-test. The exertion of the receiver operator characteristic (ROC) curve was to analyze the diagnostic value of SIRT6 and VNN1 for NS and the assessed value for the disease of child patients. The employment of logistic regression was to analyze the association of SIRT6 with VNN1 and the unpleasing prognosis of NS child patients. *P* < 0.05 was accepted as indicative of distinct differences.

## 3. Results

### 3.1. Comparison of SIRT6 and VNN1 is between the Study and the Control Groups

**Figure 1:**
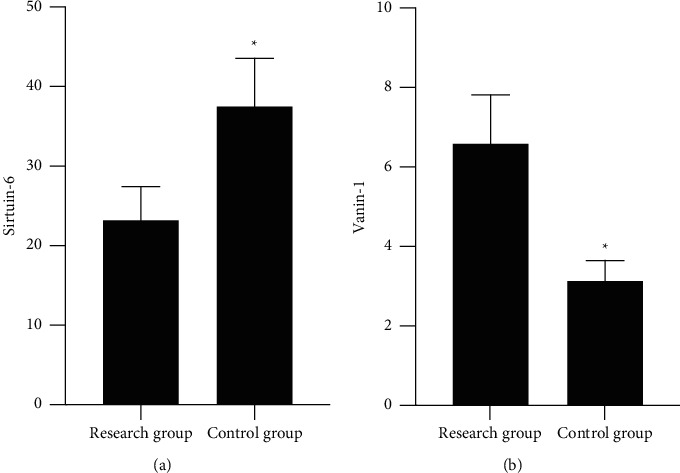
The comparison of SIRT6 and VNN1 is between the study and the control versus the study; ^*∗*^*P* < 0.05. (a) The SIRT6 in two groups. (b) The VNN1 in two groups.

SIRT6 in monocytes in the study declined versus the control, while VNN1 ascended (*P* < 0.05), as proved in Figures 1(a) and 1(b).

### 3.2. The Diagnostic Value of SIRT6 and VNN1 in NS Is Analyzed

**Figure 2:**
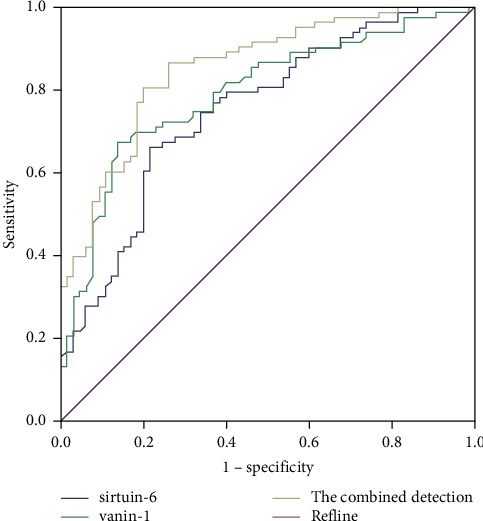
The ROC curve analysis of SIR6 and VNN1 is for the diagnosis of NS.

The AUC of SIRT6 and VNN1, jointly detecting NS, outweighed the two alone (*P* < 0.05), as proved in Table 2 and Figure 2.

### 3.3. Comparison of SIRT6 and VNN1 is between the Mild and the Severe

**Figure 3:**
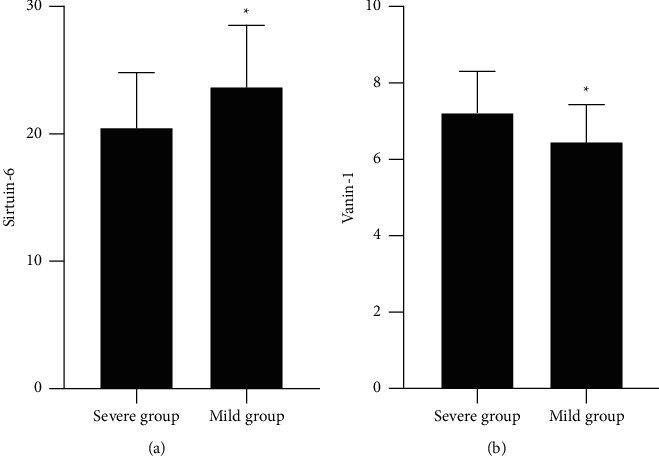
The comparison of SIRT6 and VNN1 is between the mild and the severe versus the severe, ^*∗*^*P* < 0.05.

SIRT6 in monocytes in the severe declined versus the mild, while VNN1 was elevated (*P* < 0.05), as proved in Figure 3.

### 3.4. Assessed Value of SIRT6 and VNN1 is on the Degree of Renal Function Impairment

**Figure 4:**
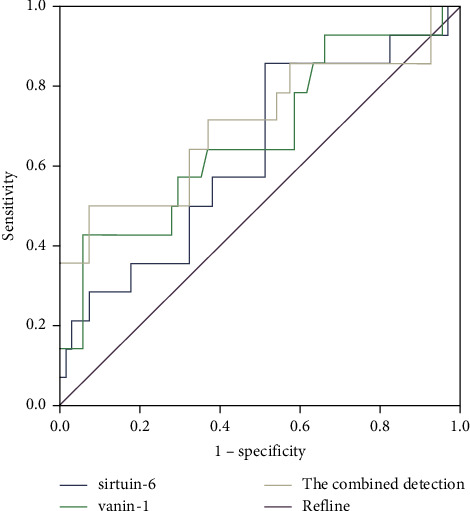
The ROC curve analysis of SIRT6 and VNN1 is to assess the degree of renal function impairment.

The AUC of SIRT6 and VNN1, jointly examining and assessing the degree of renal function impairment in NS child patients, outweighed SIRT6 alone (*P* < 0.05), as proved in Table 3 and Figure 4.

### 3.5. Comparison of SIRT6 and VNN1 is between the Good and the Unpleasing Prognosis

**Figure 5:**
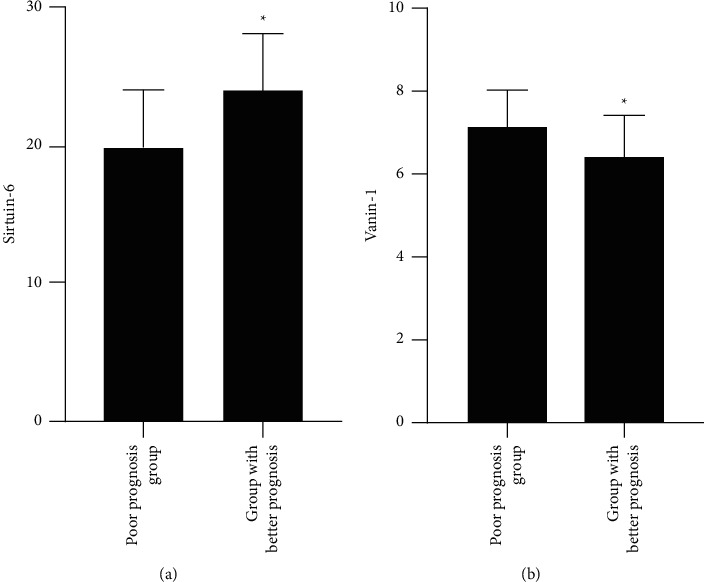
The comparison of SIRT6 and VNN1 is between the good and unpleasing prognosis versus the unpleasing prognosis; ^*∗*^*P* < 0.05.

SIRT6 in monocytes with an unpleasing prognosis was inferior than in those with a good prognosis, while VNN1 ascended (*P* < 0.05), as shown in Figure 5.

### 3.6. Assessed Value of SIRT6 and VNN1 in Prognosis Is Analyzed

**Figure 6:**
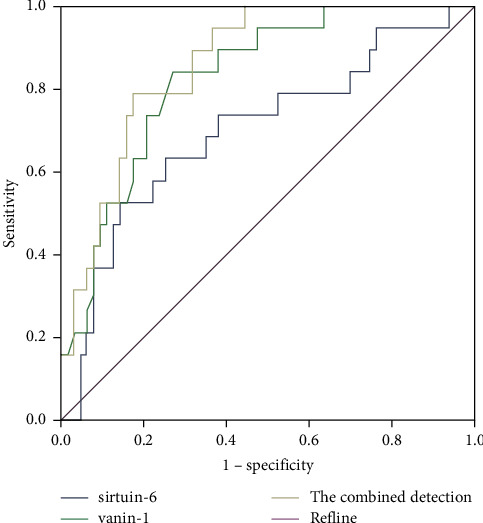
The ROC curve analysis of SIRT6 and VNN1 is conducted to predict prognosis.

The AUC of SIRT6 and VNN1, jointly detecting and assessing prognosis in NS child patients, outweighed SIRT6 alone (*P* < 0.05), as proved in Table 4 and Figure 6.

### 3.7. Correlation of SIRT6 with VNN1 is with Prognosis of NS Child Patients

Less than 21.09 in SIRT6 was the number of risk factors for the prognosis of NS child patients (*P* < 0.05), as shown in Tables 5 and 6.

## 4. Discussion

VNN1 is the critical molecule that modulates the dependent reaction of glutathione to oxidative damage in epithelial cells. Relevant studies have testified that urinary VNN1 distinctly ascends in patients with nephropathy, implying that urinary VNN1 is able to be employed as a biomarker of renal tubular injury [9, 10]. Presently, the role of SIRT6 in a variety of diseases is yet disputable, and some scholars maintain that it is a tumor suppressor modulating the occurrence and advancement of multiple tumor diseases [11, 12]. However, SIRT6 is elevated in cancers involving prostate cancer or breast cancer and is available to exert a role as an oncogene [13, 14]. Additionally, it is the same in small-cell lung cancer. SIRT6 is available to modulate glucose metabolic homeostasis via repressing glycolysis-related genes. When SIRT6 is inactivated, acetylation of glycolysis gene promoters and multiple metabolic genes are elevated, ultimately affecting the glycolysis process [15]. Relevant studies manifest the correlation of SIRT6 with diversified cell functions, involving DNA repair, antioxidant activity, cell proliferation, and mitochondrial energy homeostasis [16]. Nevertheless, cellular mitochondrial function was associated with renal function. SIRT6 is supposed to be correlated with renal dysfunction. In this study, aberrant SIRT6 and VNN1 in monocytes were verified in child patients with NS. Additionally, the study documented that the combined detection of SIRT6 and VNN1 is provided with diagnostic values for NS, so SIRT6 and VNN1 in monocytes are supposed to be applied to the initial diagnosis of NS.

The kidney is one of the most energy-demanding organs in the human body, which requires a great deal of ATP production via mitochondria to meet its physiological requirements [17]. Relevant studies testify that SIRT6 is nearly correlated to the mitochondrial function of body cells [18, 19]. SIRT6 exerts a critical role in antioxidant stress, mitochondrial substrate metabolism, and cell survival, and is able to mitigate the severity of renal tubular injury via protecting mitochondrial integrity [20, 21]. The study manifested that the AUC of SIRT6 and VNN1 jointly examining and evaluating the degree of renal function impairment in NS child patients outweighed SIRT6 alone, illustrating that the combined detection of SIRT6 and VNN1 provides an assessed value in the severity of the disease in NS child patients.

Renal injury mainly affects renal tubules, and tubular cells involve more mitochondria. It has been reported that SIRT deficiency aggravates early fibrosis in acute renal injury induced via ischemia-reperfusion in mice [22, 23]. The authors maintain that aberrant SIRT6 is supposed to exacerbate renal tissue fibrosis or critically affect the prognosis of child patients. This study documented that less than 21.09 of SIRT6 exerts a deteriorative influence on the prognosis of NS child patients, and its specific mechanism is yet unknown. Furthermore, the author maintains that this is supposed to be correlated to the fact that declined SIRT6 is available to augment the apoptosis and mitochondrial damage of renal tubular epithelial cells. Additionally, declining SIRT6 is able to aggravate the oxidative stress response and augment inflammatory damage, which is also one of the reasons for the unpleasing prognosis [24, 25]. The study stated that the AUC of the combined examination of SIRT6 and VNN1 to assess the prognosis of NS child patients outweighed SIRT6 alone, manifesting that the combined assay provided predictive value for the prognosis of NS child patients.

In short, aberrant SIRT6 and VNN1 in monocytes are proven in NS child patients, which provides diagnostic value for NS and assesses value for disease severity and prognosis.

## Figures and Tables

**Table 1:** Comparison of clinical data between the two.

Classification	The study (*n* = 83)	The control (*n* = 65)	*χ * ^2^/*t*	*P*
Gender (male/female)	51/32	44/21	0.619	0.432
Age (years)	7.14 ± 1.35	7.36 ± 1.41	0.965	0.336
24 h urine protein quantification (mg/d)	261.35 ± 50.08	115.08 ± 21.35	22.020	<0.001
Serum albumin (g/L)	19.68 ± 3.85	22.31 ± 1.89	5.049	<0.001

**Table 2:** Analysis of diagnostic value of SIRT6 and VNN1 for NS.

Indexes	Cut-off values	AUC	SE	95% CI	Specificity	Sensitivity
SIRT6	25.07	0.751	0.040	0.673∼0.830	0.663	0.785
VNN1	5.26	0.794	0.037	0.721∼0.866	0.675	0.862
Combined detection		0.854	0.031	0.794∼0.914	0.807	0.800

**Table 3:** Analysis of assessed value of SIRT6 and VNN1 on the degree of renal function impairment.

Indexes	Cut-off values	AUC	SE	95% CI	Specificity	Sensitivity
SIRT6	21.68	0.630^*∗*^	0.084	0.465∼0.795	0.602	0.778
VNN1	5.37	0.672	0.085	0.506∼0.839	0.566	0.831
Combined detection		0.705	0.090	0.528∼0.881	0.653	0.815

Versus combined detection, ^*∗*^*P* < 0.05.

**Table 4:** Analysis of assessed value of SIRT6 and VNN1 in prognosis.

Indexes	Cut-off values	AUC	SE	95% CI	Specificity	Sensitivity
SIRT6	21.09	0.699^*∗*^	0.072	0.557∼0.841	0.554	0.762
VNN1	5.54	0.829	0.049	0.733∼0.924	0.751	0.815
Combined detection		0.860	0.041	0.779∼0.942	0.792	0.846

Versus combined detection, ^*∗*^*P* < 0.05.

**Table 5:** Univariate analysis of SIRT6 and VNN1 and prognosis of NS child patients.

Indexes		The unpleasing prognosis (*n* = 19)	The good prognosis (*n* = 64)	*χ * ^2^	*P*
SIRT6	≥21.09	5	52	20.552	<0.001
	<21.09	14	12		
VNN1	≥5.54	12	11	15.456	<0.001
	<5.54	7	53		

**Table 6:** Logistic regression analysis of SIRT6 and VNN1 and prognosis of NS child patients.

Indexes	*β*	SE	Wald *χ*^2^	OR	95% CI	*P*
SIRT6	−0.155	0.078	3.949	0.856	0.735∼0.998	0.048
VNN1	1.822	0.527	2.433	2.275	0.810∼6.391	0.120
Constant	−7.141	4.260	2.810	0.001	0.000∼3.349	0.094

Value assignment: SIRT6 (21.09 or more is 1, less than 21.09 is 0); VNN1 (5.54 or more is 1, less than 5.54 is 0).

## Data Availability

The data used to support the findings of this study are available from the corresponding author upon request.
